# TOP2B: The First Thirty Years

**DOI:** 10.3390/ijms19092765

**Published:** 2018-09-14

**Authors:** Caroline A. Austin, Ka C. Lee, Rebecca L. Swan, Mushtaq M. Khazeem, Catriona M. Manville, Peter Cridland, Achim Treumann, Andrew Porter, Nick J. Morris, Ian G. Cowell

**Affiliations:** 1 Institute for Cell and Molecular Biosciences, Faculty of Medical Sciences, Newcastle University, Newcastle upon Tyne NE2 4HH, UK; larrylee585@gmail.com (K.C.L.); r.bramley@ncl.ac.uk (R.L.S.); M.M.K.Khazeem2@newcastle.ac.uk (M.M.K.); manville@rand.org (C.M.M.); peter.cridland@gmail.com (P.C.); 2 NUPPA, Newcastle University, Newcastle upon Tyne NE2 4HH, UK; achim.treumann@newcastle.ac.uk (A.T.); Andrew.Porter@newcastle.ac.uk (A.P.); 3 School of Biomedical Sciences, Faculty of Medical Sciences, Newcastle University, Newcastle upon Tyne NE2 4HH, UK; n.j.morris@ncl.ac.uk

**Keywords:** Topoisomerase II, TOP2, Cell cycle, cell biology, transcription, review

## Abstract

Type II DNA topoisomerases (EC 5.99.1.3) are enzymes that catalyse topological changes in DNA in an ATP dependent manner. Strand passage reactions involve passing one double stranded DNA duplex (transported helix) through a transient enzyme-bridged break in another (gated helix). This activity is required for a range of cellular processes including transcription. Vertebrates have two isoforms: topoisomerase IIα and β. Topoisomerase IIβ was first reported in 1987. Here we review the research on DNA topoisomerase IIβ over the 30 years since its discovery.

## 1. Discovery of DNA Topoisomerase IIβ (TOP2B) Protein

In 1987 the first report of a second type II isoform in mammalians was published by Fred Drake’s group at Smith Kline French (SKF); this isoform was called DNA topoisomerase IIβ (TOP2B) [1]. The purification of topoisomerase IIβ was from a murine P388 leukemia cell line selected for resistance to amsacrine [2]. This amsacrine resistant cell line also displayed resistance to other drugs that targeted topoisomerase II, but not to camptothecin which targets topoisomerase I. When topoisomerase II was purified from the resistant cell line and the sensitive cell line, two different sized bands were seen on SDS-PAGE gels, one of 170 KDa (p170) and the other 180 KDa (p180), but the relative amounts were different in the sensitive and resistant cell lines. Partial proteolysis with Staphylococcus V8 protease revealed different cleavage products with p170 and p180, suggesting that they were different isoforms. Antibodies were raised to each of the two purified proteins and used on western blots of cell lysates prepared by boiling immediately in SDS. The antibodies recognised specifically p170 or p180, supporting the distinct identities of the two proteins and discounting the alternative explanation that the p170 was a proteolytic fragment of the p180 (one suggestion in the field at that time).

Interestingly, two other studies published at this time also provided evidence for a second type II isoform. Joseph Holden and his team were analysing levels of topoisomerase activity and found TOP2 activity in non-proliferating tissue, which was contrary to the previous idea that only proliferating cells contain type II topoisomerase activity; they suggested this was evidence that human tissues may contain more than one form of the enzyme [3]. Joaquim Roca and Cristobal Mezquita were analysing the role of topoisomerases in chromatin changes during spermatogenesis. The presence of type II DNA topoisomerase activity in both replicating chicken testis cells and in non-replicating chicken spermatids provided evidence that there may be a second isoform of topoisomerase II in non-replicating cells [4].

## 2. Cloning the cDNA Encoding TOP2B

**Figure 1:**
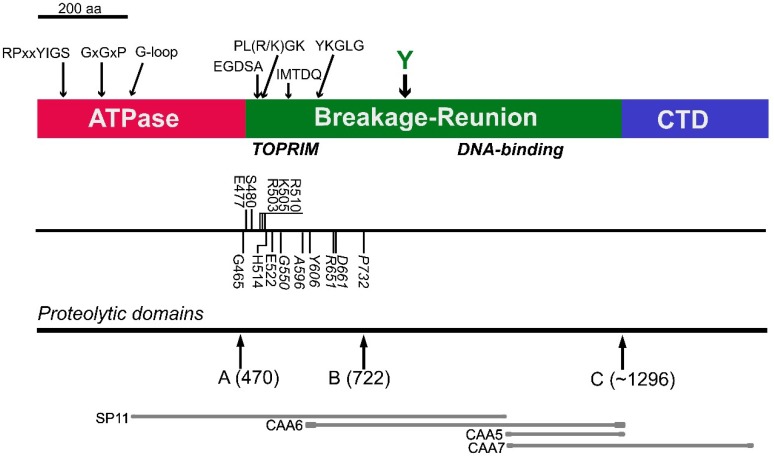
Schematic representation of the domain arrangement of human topoisomerase IIβ. The ATPase domain is shown in red, the breakage-reunion domain in green and the C-terminal domain in blue. The positions of conserved motifs are marked above. The line underneath shows the location of site-directed mutations reported by West et al. [7] (above) and mutations selected for drug resistance reported in Leontiou et al. [8] (below). The locations of inter-domain hinge regions elucidated by limited proteolysis reported in Austin et al. [9] are also shown. At the bottom of Figure 1, the locations of the initial partial cDNA clones encoding portions of TOP2B are shown [5]; SP11, and CAA5, clone CAA6, confirming that CAA5 and SP11 were part of the same gene, are also shown [10,11].

Before whole genome sequencing, obtaining coding sequences was a very time consuming process. Three groups working independently reported the cloning of partial cDNAs encoding TOP2B, using various approaches. Fred Drake and K.B. Tan’s team used a Drosophila Top2 to screen a cDNA library derived from human Raji-HN2 cells; they cloned two classes of cDNAs reported in Chung et al. [5]. One class was identical in sequence to part of the cDNA cloned for TOP2 by Monica Tsai-Pflugfelder et al. in 1988 [6]. The second class of cDNA shared 75% nucleotide identity with the other class. These were partial clones (SP1 and SP11) for the two different isoforms: topoisomerase IIα (TOP2A) and topoisomerase IIβ (TOP2B); the region encoded by clone SP11 is shown in Figure 1.

In a parallel study, a novel topoisomerase II cDNA clone was identified adjacent to the region cloned by Chung et al. [10]. Two types of clone were identified, CAA4 and CAA5; CAA4 was identical to part of the TOP2A identified by Tsai-Pflugfelder et al., in 1988 from HeLa [6] cells and CAA5 was a novel cDNA, which shared 62% identity with TOP2A and aligned with the end of the SP11 clone reported by Chung et al. [5]. The region encoded by clone CAA5 is shown in Figure 1. These clones were isolated from a human HeLa cell lambda GT10 cDNA library by screening with a small, 231 bp cDNA (CAA1) that had been isolated by expression screening using a polyclonal antisera raised to calf thymus topoisomerase II [12]. Further screening isolated larger clones (CAA6 and CAA7) that confirmed this novel cDNA was part of TOP2B. Clustal alignments of the sequences determined that TOP2A and TOP2B shared homology with topoisomerases from other species and that there were motifs conserved across all species. These conserved motifs are shown in Figure 1 [9,11]. Tsutsui et al., 1993 [13] isolated *TOP2B* cDNA clones from rat by reverse transcriptase PCR using degenerate oligonucleotides encoding regions conserved between human and Drosophila topoisomerase II. The full coding sequence for *TOP2B* was also reported by Jenkins et al., 1992 [14], using a size selected cDNA library screened by hybridisation using a PCR product generated with primers whose sequences were derived from the previously published partial clones. The chromosomal mapping revealed human topoisomerase IIα (*TOP2A*) is located on chromosome 17 [6,15] and human topoisomerase IIβ (*TOP2B*) on chromosome 3 [14,15]. These studies confirmed that *TOP2B* is a new isoform, not a splice variant, of *TOP2A.* The *TOP2B* gene was subsequently cloned and confirmed *TOP2B* was a paralog of *TOP2A* having arisen by gene duplication of *TOP2A* [16,17]. A splice variant of *TOP2B* was reported by Davies et al., in 1993 [18]. There have been several studies on *TOP2B* gene regulation; the *TOP2B* promoter lacks a canonical TATA box [19,20]. Promoter activity is regulated by nuclear factor-Y (NF-Y)- and specificity protein-1 (Sp1) [20]. TOP2B expression has also been reported to be regulated by Nurr1 [21]. *TOP2A* and *TOP2B* homologues have been identified in at least 97 species, including mice, chicken and zebrafish, and appears to be specific to vertebrates.

## 3. Protein Characterisation

TOP2A and TOP2B are paralogues that share significant sequence identity; they both have an N terminal ATPase domain, a central breakage and re-joining domain and a C-terminal domain. Limited proteolysis revealed the accessible domain boundaries; the domains and the location of the proteolytic sites are indicated in Figure 1 [9]. The domain arrangement is conserved between all type II topoisomerases, from bacterial and phage enzymes through to human enzymes. TOP2B shares 68% identity at the amino acid level with TOP2A, but this is not spread evenly through the protein. The N-terminal three quarters of the enzyme, bearing the ATPase domain and the central breakage re-joining domain, share 78% amino acid identity while the least conserved domain C-terminal domain shares only 34% amino acid identity. The divergent sequence of the C-terminal domain suggests it may play a role in the differential activities of the two isoforms both in vitro and in vivo. The domains are conserved from bacterial gyrase to human TOP2 [11]. Amino acid comparisons reveal a number of very conserved motifs, shown in Figure 1, and include RP-YIGS, G-G-P motif, the G-loop consisting of three conserved glycine’s, EGDSA, PLRGK and IMTDQDQDG motifs, and YKGL and RY where Y is the active site tyrosine [11]. Production of recombinant protein enabled studies where absolutely conserved residues were mutated. Mutation of residues E477, R503 and R505 caused loss of function in vivo, and mutation of E477 and K505 altered the magnesium optima in vitro. Residues D557, D559 and D561 are conserved in type I & II topoisomerases, and comparisons with DNA polymerases suggested that they are involved in magnesium co-ordination [7]. A number of these conserved residues, E477 and G478 in EGDSA; G504 in PLRGK; D557, D558 and G562 in IMTDQDQDG are anchor residues in the TOPRIM domain conserved between type 1A and type II topoisomerases, DnaG-type primases, OLD family nucleases and RecR proteins [22]. The crystal structure of the TOP2B core including the TOPRIM domain has been reported [23], and the core of TOP2A has also been crystallised [24]. This domain of TOP2A and TOP2B displays structural similarity between the two isoforms and with the yeast TOP2 core [25]. The structure and mechanism have been extensively reviewed, see for example [25,26,27]. The ATPase domain of TOP2A has been crystallised [28], but not that of TOP2B. There is no structural data on either TOP2A or TOP2B C-terminal domains. The C-terminal domain has least homology between the isoforms and is the site for the nuclear localisation signals and many post translational modifications including phosphorylation, sumoylation, ubiquitination, acetylation [29]. Recombinant proteins have been used to determine in vitro activities, and in vitro both isoforms exhibit strand passage activity, evidenced by decatenation and relaxation assays. Constructs with the CTDs swapped between the isoforms were used to investigate the function of the CTD in vitro [30,31]. Truncated TOP2B lacking the CTD bound to DNA most strongly, suggesting the CTD may have a negative regulatory role on DNA binding. In cells the CTDs confer isoform specificity [32,33].

## 4. Expression of TOP2B

The availability of cDNA clones to *TOP2B* and *TOP2A* allowed various groups to visualise the RNA transcripts by northern blotting, and in situ hybridisation. Northern Blotting analysis of total RNA extracted from 22 tissues in 3-month-old mice showed that the thymus expressed the highest levels of both *Top2a* and *Top2b.* For *Top2a* the spleen and bone marrow also showed high level expression. Testis and intestine expressed an intermediate level of *Top2a* and ovary, heart, breast and stomach showed low level expression. *Top2a* was not detectable in the other tissues studied. *Top2b* expression was detectable in 19 of the tissues analysed [34]. Northern blotting of RNA extracted from tissues of two-week-old rats also showed high levels of *Top2a* in spleen and thymus, but not in brain, lung, heart, liver, intestine or muscle. As seen in murine tissues, *Top2b* was also expressed more widely in rat tissues [13].

*Top2a* and *Top2b* expression was analysed in murine liver and brain before and after birth. Strikingly *Top2b* was induced more than 6 fold in the brains of newborn mice compared to embryos, but not in liver, suggesting an important role in neonatal brain [34]. Developing rat brain has also been analysed. Northern blotting on RNA extracted from rat brain showed high levels of *Top2a* in embryonic rat brain and in the cerebellum 2 days after birth, but Top2a levels were undetectable 4 weeks after birth. The pattern of expression suggested *Top2a* is only expressed in proliferating cells. In contrast, *Top2b* was present in the embryo and in the post-natal stages sampled (fetal 15 days & 19 days; postnatal 2 days, 2, 4, 12 and 32 weeks). More detailed location of transcripts was determined by in situ hybridisation of rat brain sections from postnatal day 10. *Top2a* was only seen in the proliferating regions, whereas the signal for *Top2b* was much more widespread and very intense in the cerebellum. This is shown clearly in an in situ figure in Tsutsui et al., 1991 [13].

RNA in situ in human fetal tissues (kidney, brain, intestine, liver, lung and placenta) showed differential expression of *TOP2A* and *TOP2B,* with *TOP2A* expressed most strongly in zones of proliferation [35]. In situ hybridisation of foetal cortex showed *TOP2B* expressed through the cortex [36]. This study also analysed the protein expression in human foetal brain using immunohistochemistry; this showed that TOP2A protein was present in regions of cell division and that TOP2B protein was present in both proliferative and post mitotic areas.

TOP2 protein and activity levels were analysed in rat brain. TOP2A was present during embryogenesis and up to post-natal day 1, whilst TOP2B was expressed at all ages [37]. TOP2 protein levels in murine tissues have been analysed by western blotting [38], whilst human TOP2 protein levels have been analysed by immunohistochemistry (see for example [39,40]). These studies showed TOP2A tightly associated with tissues containing proliferating cells whilst TOP2B is expressed more widely, including in post mitotic tissues. Transcriptome analysis of human tissues via the GTEx portal (www.gtexportal.org) reveals wide expression of *TOP2B* with highest expression in the cerebellum, while microarray analysis of *TOP2B* expression in primary human cells BioGPS.org) also demonstrates broad expression and highlights high expression in CD34^+^ bone marrow cells.

## 5. TOP2B In Vivo Functions

A type II topoisomerase is essential for mitosis. Both human isoforms can rescue yeast strains carrying a temperature sensitive mutation in *top2* [41,42]. However, in human cells the essential mitotic functions are carried out by *TOP2A* [43,44]. Murine embryos lacking *Top2a* terminated at the 4- or 8-cell stage, confirming that mammalian cells require *Top2a* [45]. Mice lacking *Top2b* developed in utero, but the pups died shortly after birth due to lack of innervation of the diaphragm. Motor axons failed to contact the skeletal muscle and sensory axons failed to enter the spinal cord [46]. Conditional inactivation of *Top2b* in brain tissue using a conditional Cre-*loxP* system resulted in altered brain structures and abnormal cerebral lamination. Corticogenesis was adversely affected and neurons failed to migrate normally [47]. NURR1 (nuclear receptor related 1 protein) regulates expression of *Top2b*; in *Nurr*1 knockout mice *Top2b* is downregulated [21], suggesting *Top2b* is a downstream target of *Nurr1*. A number of studies have reported that TOP2 inhibitors reduce neurite formation, and isolated neurones from *Top2b* null embryos exhibit shorter neurites than controls [48,49]. Furthermore, genes regulated by TOP2B affect neuronal survival [50]. Since *Top2b* is regulated by NURR1 and NURR1 is known to be involved in the regulation of the dopaminergic system in the brain, TOP2B may have a role in axon genesis in dopaminergic neurons. A murine model in which T*op2b* was deleted from cardiomyocytes using a Cre-*loxP* showed protection from the cardio toxic effects of doxorubicin, confirming TOP2B plays a role in doxorubicin cardiotoxicity [51].

In zebrafish *top2a* is an essential gene [52]. Maternally encoded Top2a enables early development before the mid-zygotic transition, but transcription of *top2a* is required after this point, *top2b* cannot substitute [53,54]. A zebrafish screen to detect neurite patterning errors identified a *top2b* mutant (notorious, noto) with impaired neurite targeting to the inner plexiform layer. This affects axon guidance to the retina [55]**,** further implicating*top2b* in neural development.

Non-replicating spermatids were shown to contain topoisomerase by Roca and Mezquita in 1989 [4], providing early evidence of a second type II topoisomerase. This study was ahead of its time, since more recently TOP2B has been demonstrated to have several important functions in sperm development. For example, TOP2B and PARP co-ordinate the chromatin reorganisation in developing spermatids when histone proteins are replaced by transition proteins and then by protamines [56,57,58,59,60,61,62].

## 6. TOP2B as an Anti-Cancer Drug Target

TOP2 carries out its strand passage activity by introducing a transient enzyme-bridged double-strand break in one DNA duplex segment through which a second segment is passed. Each protomer of the dimeric enzyme remains covalently linked to the 5′-end of the DSB via a phosphotyrosyl linkage during this process. The enzyme subsequently religates the break and is released. Human type II DNA topoisomerases are the molecular target for a number of drugs used in cancer chemotherapy. These drugs, referred to as TOP2 poisons, inhibit the religation step, and thus stabilise the normally transient enzyme-bridged DNA DSB that occurs during the TOP2 strand-passage reaction. These TOP2-DNA covalent complexes are cytotoxic and in sufficient quantity result in loss of cell viability. Clinically relevant TOP2 poisons include doxorubicin, epirubicin, daunorubicin, idarubicin, mitoxantrone, etoposide and mAMSA. There are extensive numbers of reviews on this topic, see, for example [63,64,65]. Interestingly, new combinations including these drugs are still being tested in clinical trials. For example, a recent trial found that mAMSA could reduce relapse incidence in AML-patients [66].

The discovery of TOP2B raised the question of whether TOP2A, TOP2B or both isoforms are targeted by different TOP2 poisons. However, a series of studies demonstrated that both TOP2B and TOP2A are targeted by TOP2 poisons. For example, in clongenic assays, murine embryo fibroblasts lacking *Top2b* were less sensitive to mAMSA than wild type cells and also demonstrated some resistance to mitoxantrone and etoposide [67]. This was confirmed in human cells lacking *TOP2B*, where *TOP2B* null cells showed the greatest resistance to mAMSA and mitoxantrone, and also showed resistance to etoposide and doxorubicin. Notably, in this study *TOP2B^−/−^* null cells were significantly less sensitive to mAMSA and mitoxantrone than were *TOP2A^+/−^* heterozygotes, while for etoposide the *TOP2A,* heterozygotes demonstrated the greatest resistance [68,69]. Further evidence for targeting of TOP2B by TOP2 poisons was provided using a yeast ts top2 system expressing functional human TOP2B. Human TOP2B complements the ts yeast top2 at the non-permissive temperature and this allows the selection of drug resistance mutants in the human enzyme in vivo. Nine mutated proteins were selected from a randomly mutated pool that conferred drug resistance to acridines, including mAMSA, confirming TOP2B is a target for acridines [8,70,71,72].

Using an isoform-specific microscopy based immunoassay (trapped in agarose DNA immunostaining or TARDIS assay) [73,74] to detect TOP2-DNA covalent complexes, we showed that etoposide induces complexes with both TOP2 isoforms, as does mAMSA and mitoxantrone [75,76,77]. The half-lives of the complexes in cells can be determined using the TARDIS assay. Etoposide-stabilised complexes have half-lives of 30 min for TOP2A and 15 min for TOP2B in human CCRF-CEM cells [76], whilst in MEFs the etoposide half-life for Top2a is 40 min and for Top2b is 20 min. For mAMSA the half-life for both Top2a and Top2b is 15 min and mitoxantrone complexes are much longer lived, with a half-life of 10 h for Top2a and 6 h for Top2b [76]. The TARDIS assay is a microscopy-based method that allows the detection and quantification of TOP2-DNA covalent complex levels in individual cells. An alternative isoform specific assay is the ICE-assay, which involves ultra-centrifugation to separate the protein–DNA complexes followed by blotting the collected fractions on a slot blot and probing with isoform specific antibodies. This method also shows complexes with both TOP2 isoforms [68].

While they are effective and widely used anti-cancer agents, TOP2 poisons are also associated with therapy-related acute leukaemia, in particular acute myeloid leukaemia (t-AML) [78,79,80]. These t-AML cases often exhibit one of a set of recurrent chromosome translocations that drive the development of the leukaemia, most frequently involving the *MLL/KMT2A* gene at 11q23, t(15,17)(*PML-RARA*), t(8,21)(*RUNX1-ETO*), or inv(16) (*CBFB-MYH11*) [79,81]. This raises the question of the relative contribution of TOP2A versus TOP2B in the aetiology of TOP2 poison-induced t-AML. While in principle either or both TOP2 isoforms could contribute to the induction of leukaemia-causing translocations via DNA cleavage at multiple loci followed by mis-repair, several lines of evidence point to TOP2B as the major contributor. For example, TOP2B is required for most etoposide-induced chromosome breaks at the *MLL/KMT2A* and *RUNX1* loci using DNA FISH in a human cell line model [82,83], top2b is required for efficient etoposide-induced recombination in a plasmid integration assay [84] and TOP2B is implicated in androgen-induced chromosome rearrangements in prostate cancer [85]. In addition, translocation partner genes such as *KMT2A* and *AF9* or *RUNX1* and *ETO* are closely juxtaposed in a fraction of cells, and this may be influenced by TOP2B [82,83]. This led to a model for the aetiology of TOP2 poison induced leukaemia where chromosome translocations are facilitated by co-localization of genes within common transcription factories, combined with TOP2B-mediated transcription-linked DNA breaks [78,82,83,86]. Furthermore, a proportion of TOP2B co-localises at conserved genomic locations with CTCF and members of the cohesin complex including RAD21 [87], presumably to resolve DNA topological problems. ChIP-seq combined with end-seq analysis demonstrated that this generates fragile sites in the genome that can contribute chromosome translocation [87,88,89,90]. Notably, t-AML patient associated *MLL/KMT2A* translocation breakpoints are enriched within a 1 kb region of intron 11 containing at one end a CTCF binding site [82,89].

## 7. TOP2B Cell Biology–Cell Cycle Expression

In contrast to its essential role in vertebrate development as observed in mice and zebrafish, in mammalian cell lines, TOP2B is not essential for cell viability [91,92]. There are cell cycle differences in TOP2A and TOP2B protein levels. In murine NIH-3T3 cells Top2a decreases 5.5 fold when cells plateau, whilst Top2b levels decrease by only 20% [93]. NIH-3T3 cells were synchronised by serum starvation to analyse the effect of the cell cycle on Top2 protein levels; this demonstrated that Top2a was highest in S phase and G2/M and Top2b was present in all phases of the cell cycle [94]. Studies on five human leukaemia cell lines (K562, Raji, Molt 4, CCRF-CEM and Jurkat) determined that the two isoforms had differing salt extractabilities, and this was cell line dependent with the optimal level varying between 1 and 1.9 M sodium chloride. The optimal salt extractability was as efficient as DNase I/RNase A digestion and SDS solubilisation [95]. TOP2 levels were quantified by western blotting with a standard curve of purified recombinant TOP2A or TOP2B and expressed as monomers per cell. Exponentially growing K562 cells contained 440,000 monomers of TOP2B and 570,000 monomers of TOP2A. Exponentially growing Raji cells were compared to confluent cultures. Exponentially growing cells contained 720,000 monomers of TOP2A and 1280,000 monomers of TOP2B. In contrast the confluent cells contained 300,000 monomers of TOP2B but only 60,000 monomers of TOP2A. In peripheral blood lymphocytes only TOP2B was detectable (290,000 monomers), consistent with differentiated cells lacking TOP2A, and in ALL patient blasts only TOP2B was detectable [95]. This is consistent with the majority of blasts being non-proliferating [96,97].

Proliferation dependent topoisomerase II content has been reported to be a determinant of topoisomerase II mediated drug action, with topoisomerase levels being higher in log phase cells than in quiescent plateau phase cells from several species. These studies included cell lines derived from mice, Chinese hamster and human; cycling cells were more sensitive to drugs targeting DNA topoisomerase than quiescent plateau phase cells [98,99,100,101,102].

**Figure 2:**
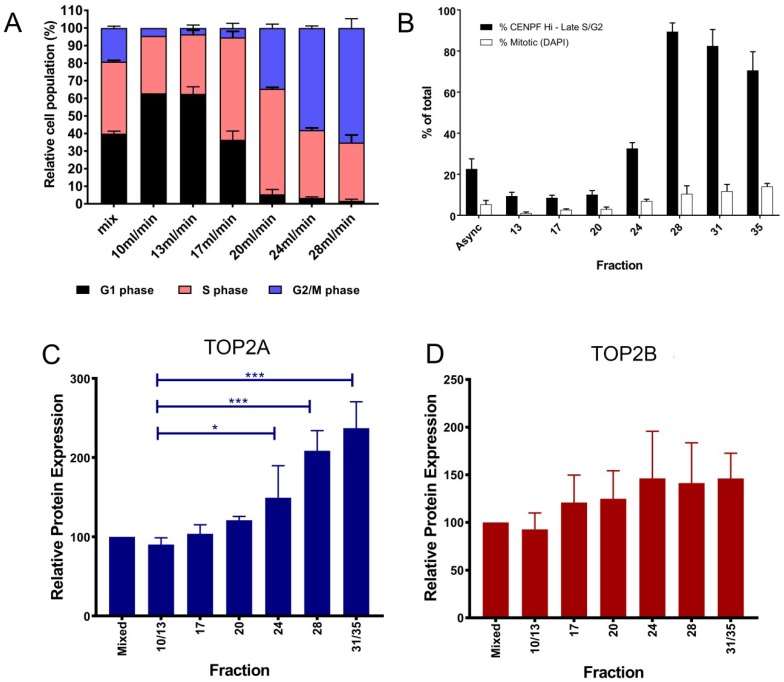
Elutriation of K562 cells separates the cells according to size. (**A**) FACS analysis (Flowjo) of each elutriation fraction showing the percentage of G1, S or G2/M phase cells. (**B**) Immunofluorescence analysis of each elutriation fraction showing the percentage of each fraction in lateS/G2/M determined by CENPF immunofluorescence and the percentage of mitotic cells determined by DAPI staining. (**C,D**) TOP2A and TOP2B protein level per cell was determined by quantitative immunofluorescence [77], the data shown represent the mean ± SEM of median values obtained from three replicate experiments. Statistical analysis was carried out using one way ANOVA (* *p* < 0.05; *** *p* < 0.0001).

We present new data investigating drug targeting and cytotoxicity on rapidly proliferating K562 cells that have not been perturbed by serum starvation, separated by size using centrifugal elutriation. The buffer flow rate determines the size of cells eluted from the rotor. Cells are loaded into the chamber at low flow rate and as the flow rate is increased cells start to elute. Smallest cells (G1 phase cells) are eluted at lower flow rates than larger cells (G2/M phase cells); fractions collected at the different flow rates had different proportions of each cell cycle phase. As expected, the first fractions eluted were enriched for G1 cells compared to the starting cell population and contained fewer G2/M cells; the next fraction (17 mL/min) had more S-phase cells and fewer G2/M cells than the starting population, and the later fractions were enriched for G2/M cells, with few or no G1 cells. All the fractions contained S phase cells, but the percentage of S phase varied between 33% and 60%, with 13 mL/min having the lowest percentage of S phase cells and 20 mL/min having the highest percentage of S phase cells (Figure 2A).

The separated cells were then analysed in a range of ways. FACS analysis was used to determine DNA content, which is an indicator of cell cycle position, and also by immunofluorescence using two cell cycle markers (CENPF and H3Ser10P). G1 cells express low levels of CENPF and low phospho-H3Ser10, while late S and G2/M cells contain increasing levels of CENPF, with highest levels in G2/M cells (Figure 2B), and condensing/condensed mitotic chromosomes are decorated with phospho-H3Ser10 [103,104]. TOP2A and TOP2B protein levels were determined on a single cell basis by quantitative immunofluorescence for each fraction. In K562 cells the levels of TOP2A increased significantly as the proportion of cells in G2/M phase increased in fractions collected at 24, 28 and 31/35 mL/min (Figure 2C) whilst TOP2B did not vary significantly between the fractions (Figure 2D). This is consistent with previous studies which showed TOP2A levels were highest in cells in G2/M phase.

**Figure 3:**
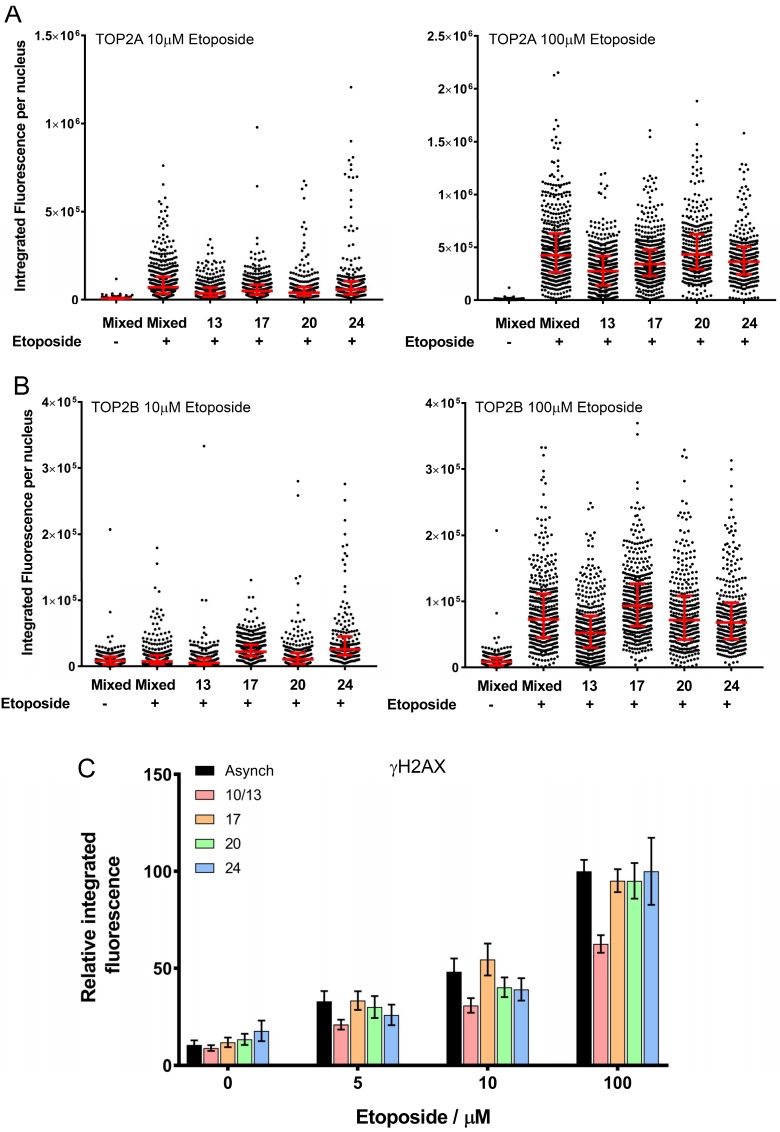
TOP2 covalent complex levels determined by trapped in agarose DNA immunostaining (TARDIS) and γH2AX integrated fluorescence. Quantification of etoposide-induced TOP2A and TOP2B covalent-DNA complexes, (**A**,**B**, respectively). Cells from each elutriated fraction were treated with etoposide (10 or 100 μM) and TOP2-DNA covalent complexes were detected and quantified by TARDIS analysis [73,74,75,77]. The data are shown as scattergrams, derived from a single representative experiment. Bars represent the median and interquartile range. (**C**) Cells from each elutriated fraction were treated with etoposide (5, 10 or 100 μM) or solvent (DMSO) and analysed by γH2AX immunofluorescence; the level of γH2AX integrated fluorescence was determined by quantitative immunofluorescence [77]. The data are shown as means of the medians obtained from three replicate experiments.

To determine if the K562 cell response to TOP2 drug targeting varied with cell cycle phase, we took the starting mixed culture and fractions collected at 10, 13, 17, 20, 24 mL/min and treated them with the TOP2 targeting drug etoposide for one hour (it was not possible to test the 28 mL/min & 31/35 mL/min fractions as too few cells were present in these fractions). Using the trapped in agarose DNA immunostaining (TARDIS) method we were able to detect TOP2A and TOP2B covalent complexes in all the fractions following exposure to 100 µM etoposide for one hour. For TOP2A, complexes were also efficiently formed in cells from each fraction following exposure to 10 µM etoposide; for TOP2B 10 µM etoposide resulted in a close to background signal for each fraction. An example of the data from a single elutriation experiment is shown in Figure 3A,B. Combined data from three independent elutriations revealed no significant difference in the formation of TOP2A or TOP2B complexes between the fractions or compared to the starting mixed population. To determine whether cell-cycle position affected etoposide-induced DSB formation, γH2AX quantitative immunofluorescence was used. γH2AX signal was seen in all fractions following exposure to 5, 10 or 100 µM etoposide for one hour, and the signal increased with etoposide concentration. Compared to the starting mixed population, two-way ANOVA of all three doses and all fractions tested indicted that the 10/13 mL fraction had lower levels of γH2AX integrated fluorescence. The 10/13 mL fraction contained fewer S phase cells than the starting population or the other four fractions tested and had the highest level of G1 cells (Figure 3C).

**Figure 4:**
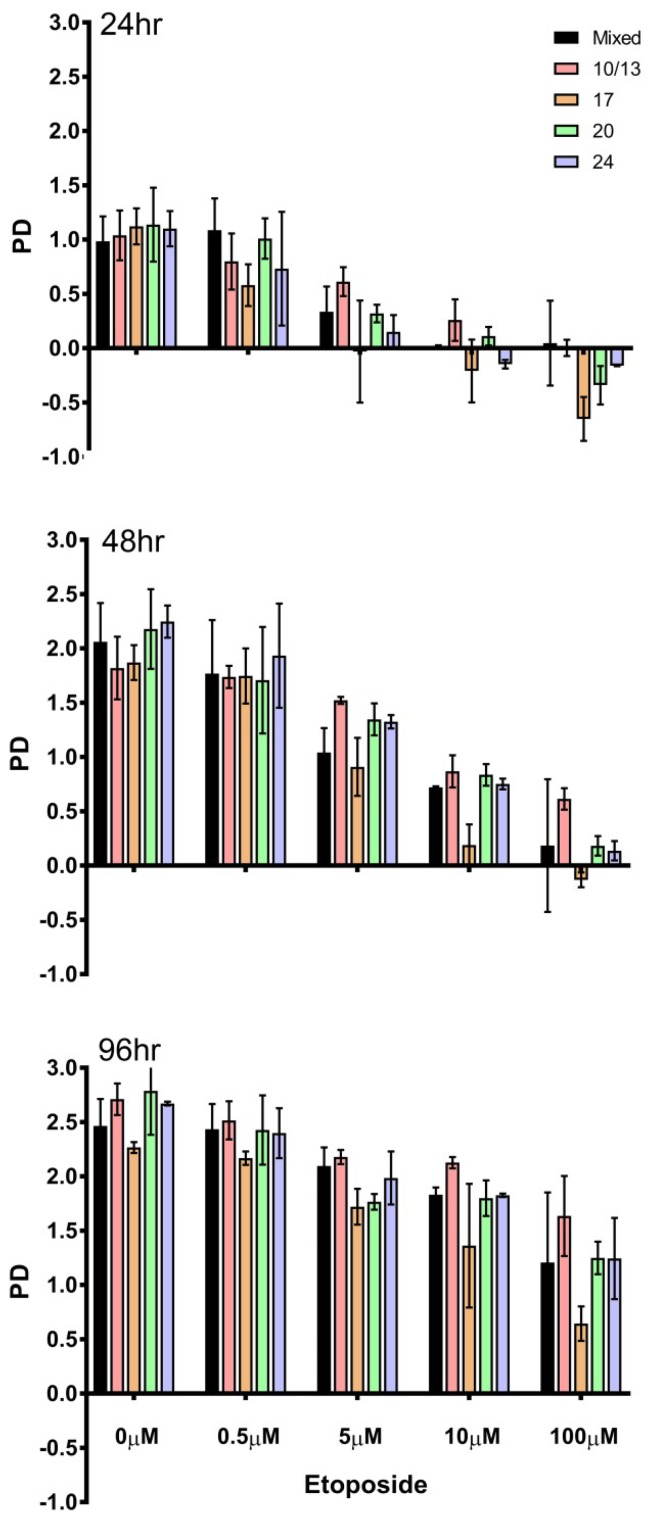
Drug sensitivity of the starting mixed population and the elutriated fractions. Cells from each elutriated fraction were treated with etoposide (0–100 μM) for 1 h. Cells were then replated in fresh medium and were counted after 24, 48 and 96 h. Population doublings were calculated according to the formula PD = Log_10_(cell count/initial cell count)/Log_10_ (2). Data represent the mean ± SEM derived from duplicate elutriation experiments.

Cytotoxicity assays were carried out on the starting mixed population and on the 10, 13, 17, 20, 24 mL/min fractions. Cells were treated for 1 h with etoposide and then placed in drug-free medium for 24, 48 or 96 h. Cell counts were analysed by population doubling. Etoposide concentrations of 0.5, 5, 10 or 100 µM were used. Figure 4 shows the population doubling 24, 48 or 96 h after a 1-h drug exposure. Population doubling was similar in the mixed population and all the fractions tested following exposure to 0.5 µM etoposide for one hour followed by growth in drug free media. Indeed, after 0.5 µM for 1 h, there seemed little difference in population doubling compared to the untreated fractions. Following exposure to 10 or 100 µM for one hour followed by growth in drug free media for 24 or 48 h, the population doubling was noticeably reduced, in some cases dropping below 0, indicating cytotoxicity and loss of cells. Compared to the starting mixed population the G_1_/early S-enriched 17 mL/min fraction was most affected. After 96 h in culture, there appeared little difference apart from fraction 17 that had been exposed to 100 µM etoposide.

In summary, for non-perturbed proliferating K562 cells separated into fractions based on size and cell cycle phase prior to exposure to etoposide for one hour, no differences in the level of topoisomerase II covalent complexes were seen in the different fractions (Figure 2A,B). Fluorescence intensity for gamma H2AX signal only differed in the 10/13 mL/min fractions which contained the most G1 phase cells (Figure 2C). In these fractions the gamma H2AX signal was lower, suggesting fewer double strand breaks were formed when there were fewer S phase cells. Minimal difference in cytotoxicity was seen with 0.5 µM etoposide when comparing the fractions with the starting population, but with higher concentrations the most sensitive fraction was that collected at 17 mL/min, containing early S phase cells. This indicates that early S phase cells are more sensitive to etoposide, consistent with observations in previous studies in cells synchronised with serum starvation [98,99,100,101,102]. However, since the levels of TOP2-covalent complexes were similar in all fractions tested (Figure 2A,B), events downstream of complex formation, such as replication fork collision, may be responsible for the increased sensitivity to etoposide in early S phase cells, reviewed in Yan et al., 2016 [105].

## 8. Subcellular Location of TOP2B Protein

TOP2 is predominantly nuclear; the C-terminal domain of TOP2A and TOP2B contain nuclear localisation signals [106,107] and both isoforms have nuclear export signals [108,109]. The interphase subcellular location of TOP2B has been the topic of a number of studies. Zini et al. reported that TOP2B was nucleolar [110], whilst another group found Top2b in both the nucleoplasm and nucleolus, and frequently associated with heterochromatin [111] and a third study suggested TOP2B was excluded from the nucleolus [112]. Chaly and Brown analysed the subcellular location of TOP2A and TOP2B under different fixation conditions with a number of antibodies. The conclusion of this study was that TOP2B was present largely in the nucleoplasm with some distribution in the nucleoli which was variable depending on antibody and fixation condition [113]. Studies with antisera raised to TOP2B CTD and using TOP2B CTD galactosidase tagged constructs in HeLa and monkey COS7 cells showed nucleoplasmic staining with no evidence for significant nucleolar enrichment [114]. Onoda et al., 2014 [115] show shuttling of TOP2B between the nucleolus and the nucleoplasm, and suggest that the enzyme is active when in the nucleoplasm. Cowell et al., 2011 demonstrated that Top2b is enriched in heterochromatic regions in mouse epithelial cells and relocates to give a pan nuclear staining upon treatment with the histone deacetylase inhibitor TSA [116], treatment which also causes loss of HP1 from heterochromatin [117]. Christensen et al., 2002, using photo bleaching, demonstrated that TOP2A and TOP2B were mobile within the nucleus [118]. Cell lysis conditions can affect the distribution of TOP2 in immunofluorescence localisation studies, and hypotonic cell lysis produced an axial pattern. During mitosis most TOP2B dissociates from chromatin, whilst most TOP2A remains chromosome bound [112,119] and the mitotic functions of TOP2A cannot be carried out by TOP2B [43]. However, some studies also find TOP2B is associated with mitotic chromosomes [120,121]. In addition, two studies have indicated the presence of Top2b in mitochondria using mass spectroscopy, the first in 2003 [122] and the most recent in 2014 [123] where Top2a was also detected in mitochondria.

## 9. Protein–Protein Interactions

A wide range of approaches have been used to identify protein interaction partners of TOP2B. One of the first proteins identified as an interacting protein was CD3 epsilon; the cytoplasmic region of CD3 epsilon was used as bait to screen an expression library and it bound to the C-terminal domain of TOP2B [124]. The TOP2B interacting protein, DNA topoisomerase IIB binding protein one (TOPBP1) was identified in a yeast two hybrid screen with the C-terminal part of TOP2B (1143–1621) as bait [125]. UBC9, a SUMO1 conjugating enzyme, was identified as a TOP2B interacting partner via a 2 hybrid screen [126]. Phosphorylation of TOP2B by a range of kinases has been reported including CKII, PKC, PLK, Aurora B, cyclin B/cdc2 and MAPK [127,128,129,130,131]. Direct interaction with TOP2B has been reported for a number of kinases. TOP2B was pulled out when ERK1 was used as bait in a protein expression screen, clone S64 encoded residues 1398–1592 of TOP2B [132]. Using a combination of site directed mutagenesis and mass spectroscopy, three sites that could be phosphorylated in vitro by ERK1 were identified between residues 1398 and 1592: Serine 1419, Serine 1445 and Serine 1471 [133]. Other interacting kinases include Protein Kinase Cζ [134] and Casein Kinase IIβ [135]. TOP2B has been shown to interact with tumour suppressor proteins p53 [136,137] and pRB [138,139]. To map the pRB interaction a yeast two hybrid system was used. This showed that the C-terminal domain of TOP2B interacted with the A box of pRB, and GST fusion protein pulldowns indicated the last 116 amino acid residues of TOP2B were sufficient for this interaction [139]. Histone deacetylates HDAC1 & HDAC2 have also been shown to interact directly with TOP2B [140]. TOP2B has also been reported to be present within several complexes involved in ligand mediated transcriptional regulation, these include an RARB/TOP2B repressor complex [141], the Groucho/TLE1 repressor complex containing PARP and TOP2B [142] and a complex containing TOP2B, PARP, DNA-PK and KU antigen [143]. Wong et al., 2009 also found TOP2B to be a component of a transcriptional complex containing DNA-PK and KU antigen that is recruited to the fatty acid synthase (FAS) gene in response to insulin [144]. An interaction between KU and TOP2B was also reported by Matheos et al., 2002 [145]. Via programmes such as STRING (https://string-db.org/) [146] it is possible to view a range of interactions as they are reported.

Most recently, biotin ID mapping has been used to identify proteins interacting with TOP2B. A biotin ligase TOP2B fusion protein was expressed in HeLa cells and proteins in close proximity to TOP2B become biotinylated. They were then isolated on streptavidin beads and identified by mass spectrometry. This study reported 25 high confidence interacting partners for TOP2B (TOP2A, TOP1, CTCF, SMC1A, PDS5B, STAG1, STAG2, PDS5A, NIPBL, RAD21, HMGA1, YY1, ZNF451, ZNF512, ZNF362, DDX18, DDX31, PHF2, SDAD1, CBX8, LRIF1, HDGF, SRBD1, RRP15, MORC2) [87]. Interestingly recent mass spec analysis of the calf thymus protein purified in 1987 [147] confirmed the TOP2 purified from calf thymus contained both TOP2A and TOP2B and it co-purified with three proteins reported to interact with TOP2B via biotin ID mapping (TOP1, DDX18, SMC1A) [87] and with proteins reported to functionally interact with TOP2B such as PARP1.

## 10. Transcriptional Roles of TOP2B

DNA topoisomerases are required to relieve the topological stress during transcription, as detailed in the twin domain supercoiling model [148]. Both type II isoforms TOP2A and TOP2B have been reported to be involved in transcriptional regulation; Top2a appears to be required for gene regulation and priming in embryonic stem cells whilst a switch to Top2b occurs during differentiation [50,149]. TOP2B has been reported to mediate ligand-mediated transcription via a mechanism that introduces a double strand break [85,141,143,150,151,152]. A number of groups have reported TOP2B ChIP-seq studies, the first reported by Sano et al. in 2008, using rat cells [153]. More recent studies have reported ChIP-seq from human MCF7 cells [154], primary mouse B cells [89], mouse liver [87] and mouse brain [155,156]. Chip-seq analyses in humans [154] and mouse [87] identify transcription factor binding sites within the peak sequences associated with topoisomerase IIB; these included CTCF, SP1, EGR1, PAX5, and TFAP2. Furthermore, as described above, Uusküla-Reimand et al. [87] also showed that TOP2B is associated with CTCF and members of the cohesin complex at domain boundaries. In addition, Canela et al. [89] demonstrated that loop anchor regions bound by cohesin and CTCF are sensitive to etoposide-induced DSBs identified by end-seq; these breaks are co-incident with TOP2 ChIP peaks suggesting they are mediated by TOP2, probably TOP2B. This is consistent with a role for topoisomerases and transcription in mediating domains of supercoiling flanked by GC-AT boundaries and CTCF insulator protein-binding sites [157]. Topoisomerases have been reported to be particularly important for the transcription of long genes [158,159,160].

## 11. Conclusions

The first report of topoisomerase IIβ protein was in 1987; that it was encoded by a separate gene was subsequently confirmed by cloning of the cDNA and mapping the gene location. Research in the first 10 years after TOP2B was reviewed in Austin and Marsh 1998 [29]. Subsequent research elucidated the biochemistry of this protein via site directed mutagenesis and crystallography of the enzyme core breakage reunion domain. Protein–protein interactions reveal that TOP2B interacts with various kinases responsible for phosphorylation; the cellular function of post translational modifications of TOP2B is an open question. TOP2B is associated with TOP2A in biotin ID mapping studies, raising the question of the presence and role of TOP2A/B heterodimers in cells. The murine knockout reported in 2000 demonstrated its importance for neural development. Studies more recently have demonstrated the importance of TOP2B in transcriptional regulation and genome organisation. Nevertheless, the detailed molecular mechanism of transcriptional regulation by TOP2B remains to be elucidated. A murine knockout in cardiomyocytes implicated TOP2B in cardiotoxicity [51]. TOP2B has also been implicated in the development of therapy related leukaemia [78,82,83]. The observations have led to the suggestion that drugs selectively targeting TOP2A may have clinical advantages; further work will be needed to determine if this is correct.

## Abbreviations

CTCF	CCCTC-binding factor
CTD	C terminal domain
DSB	Double strand DNA break
t-AML	Therapy-related acute myeloid leukaemia
TARDIS	Trapped in agarose DNA immunostaining
TOP2A	DNA topoisomerase IIα
TOP2B	DNA topoisomerase IIβ
TOP2	DNA topoisomerase II
ts	Temperature sensitive
